# Raman spectroscopy and its urological applications

**DOI:** 10.4103/0970-1591.39550

**Published:** 2008

**Authors:** Vishwanath S. Hanchanale, Amrith R. Rao, Sakti Das

**Affiliations:** Department of Urology, Leighton Hospital, Crewe, UK; 1 Wexham Park Hospital, Slough, UK; 2 University of California School of Medicine, Los Angeles, CA, USA

**Keywords:** Applications, Raman spectroscopy, review, urology

## Abstract

**Purpose:**

The Raman spectroscopic technology can be utilized for the detection of changes occurring at the molecular level during the pathological transformation of the tissue. The potential of its use in urology is still in its infancy and increasing utility of this technology will transform noninvasive tissue diagnosis. The Nobel laureate, Sir C.V. Raman is credited for the discovery of the principles of Raman spectroscopy.

**Materials and Methods:**

Applications of Raman spectroscopy in the bladder, renal, prostate, and other urological disorders were gathered from Medline and abstracts from recent international urological meetings. Current status and future directions of Raman spectroscopy in urology were also reviewed.

**Results:**

Raman spectroscopic technology is used to interrogate biological tissues. The potential use of this technology in urology has shown encouraging results in the *in vitro* diagnosis and grading of cancers of the bladder and the prostate. Raman microprobes have been used for the characterization and identification of renal lithiasis. Technology may be available for the urologists to determine the margin status intraoperatively during partial nephrectomy and radical prostatectomy. The future would see the development of optical fiber probes to incorporate them into catheters, endoscopes, and laparoscopes that will enable the urologist to obtain information during the operation.

**Conclusion:**

Raman spectroscopy is an exciting tool for real-time diagnosis and *in vivo* evaluation of living tissue. The potential applications of Raman spectroscopy may herald a new future in the management of various malignant, premalignant, and other benign conditions in urology.

## INTRODUCTION

Optical diagnostics is a rapidly evolving technology that has potential to improve the traditional methods of diagnosis. The principle of these techniques is to detect a change in the nature of light when it interacts with the tissue. Potential advantages of this technology over traditional methods are reduction of inter or intraobserver bias in interpretation of pathological findings and real-time application via minimally invasive endoscopic routes. Commonly used optical diagnostics are Raman spectroscopy, fluorescence spectroscopy, elastic scattering spectroscopy, and optical coherence tomography.

Raman spectroscopy relies on the principle of inelastic scattering of light photons on interaction with biological tissue. Fluorescence spectroscopy acts by detecting the fluorescent properties of normal and pathological tissues. As the name suggests, elastic scattering spectroscopy detects photons that have been elastically scattered or reflected. Optical coherence tomography provides morphological information on the tissue under investigation by producing high-resolution, cross-sectional tomographic images.

**Figure 1:**
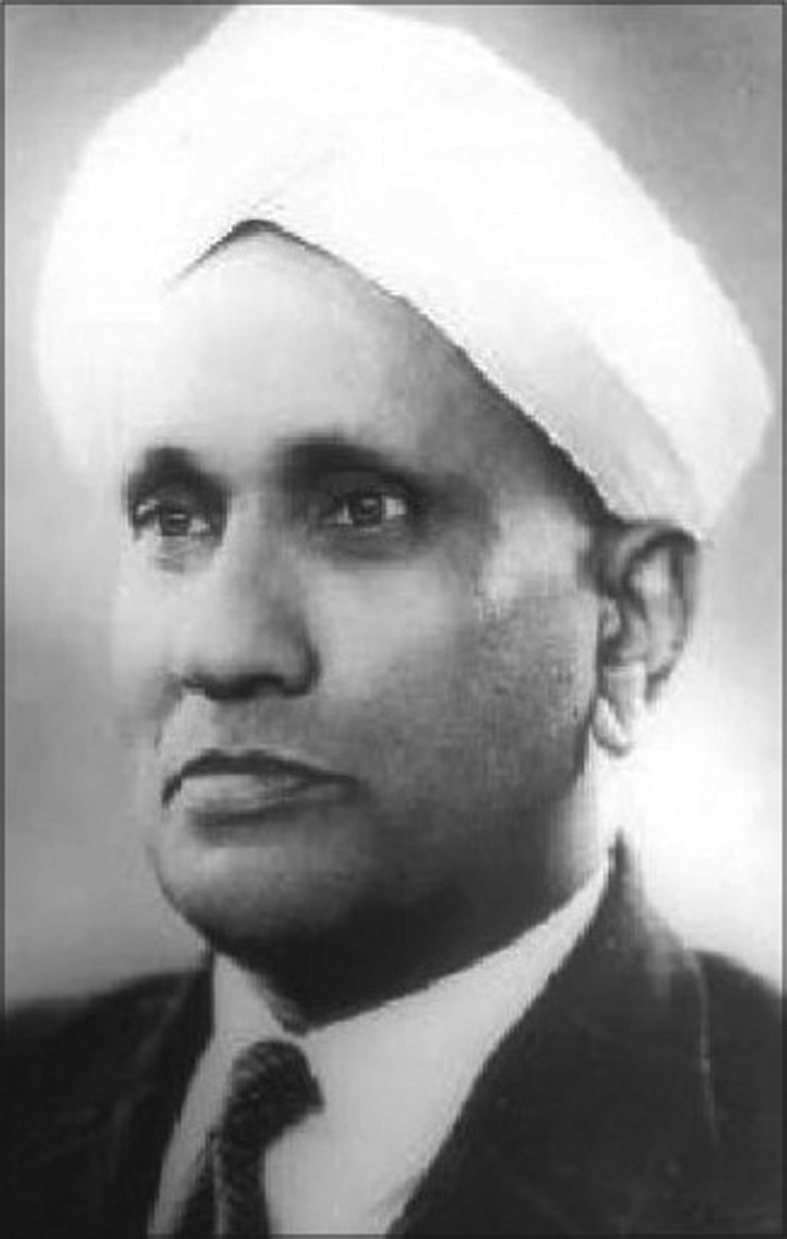
Nobel laureate, Sir C.V. Raman

Raman spectroscopy can measure the spectra for each biological molecule and this can facilitate to find the exact molecular composition of the tissue. As a nondestructive method of measuring these changes, it has the potential for real-time diagnosis during urological interventions. The Indian Genius, Sir Chandrashekhar V. Raman received the Nobel Prize in 1930 for the discovery of the principles of spectroscopy [Figure 1].

## MATERIALS AND METHODS

Articles pertaining to the life and work of the Nobel laureate Sir C.V. Raman were reviewed. Mechanics of Raman spectroscopy and its application in medical field were gathered from scientific manuscripts. Applications of Raman spectroscopy in the bladder, renal, prostate, and other urological disorders were gathered from Medline and recent urological meeting abstracts to assess the current status and explore future directions of Raman spectroscopy in urology.

## RESULTS

Tissue biopsy and histological examinations are gold standard for cancer diagnosis. The few pitfalls of this standard method include false negative results, subjective discrepancies, and complications associated with obtaining the tissue for diagnosis. Raman spectroscopy is an emerging technology to overcome these problems and can potentially become an objective, noninvasive tool for real-time determination of pathological state of tissues.

Indian physicist Sir C. V. Raman was the first Asian scientist to be awarded the Nobel Prize for his discovery on the scattering of light and Raman effect. Raman effect named in honor of C.V. Raman gave rise to Raman spectroscopy - a technology for the analysis of molecular structure. Life and work of Sir C. V. Raman and the discovery of Raman spectroscopy has been reviewed recently.[1]

**Figure 2:**
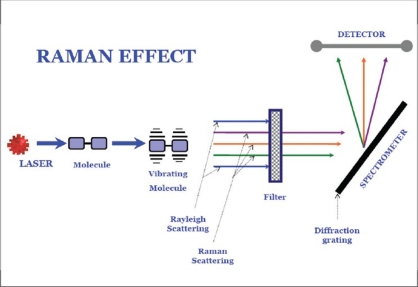
Raman effect

**Figure 3:**
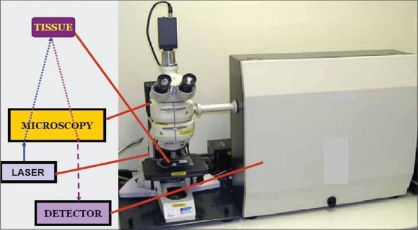
Renishaw Raman system

Raman spectroscopy is an optical technique that uses molecular-specific, inelastic scattering of light photons to interrogate biological tissues.[2] On illumination of tissue with light, photons interact with the intramolecular bonds present; the photon then donates energy to or receives energy from the bond, producing a change in the bond's vibrational state (partial quantum state). There are then emission wavelengths of equal frequency as the incoming wavelengths such that there is no net change in energy between the light and the substance. Such light wavelengths are said to be elastically scattered in a process known as Rayleigh scattering.[3] Rarely, the molecule absorbing the incoming wavelength's energy emits a wavelength of a different frequency (inelastic scattering) known as the ‘Raman shift’ [Figure 2]. Raman shift is nothing but this change in the photon energy and is measured in wavenumbers. The Raman spectrum is a direct function of the molecular composition of tissue and that can give objective picture of the pathology. Raman frequency shifts are conventionally measured in wave number (cm^−1^), a unit relating to the change in vibrational energy of the scattering molecule to the change in frequency of the scattered light. Each cm^−1^ is equivalent to 30,000 MHz, which is 10,000 times smaller than the frequency of light.[4] The incident light is often referred to as “excitation light” and in Raman spectroscopy it can be ultraviolet (UV), visible, and infrared light. UV excitation reduces the fluorescence noise that the incident light can elicit from the tissue. However, the penetration of UV light is in microns, and is mutagenic, therefore is suitable for *in vitro* studies. Visible light again has the advantage to study individual cells *in vitro*. However, it has the disadvantage of bringing out strong fluorescence on the background, thus interfering with the spectra obtained. To reduce the intrinsic tissue fluroscence, near infrared excitation using LASERS (785 nm diodes and 1064 nm Nd:YAG are commonly used) has become popular to study biological tissues. There are four main types of Raman spectroscopy in use today: surface-enhanced Raman spectroscopy (SERS), resonance Raman spectroscopy (RRS), confocal Raman microspectroscopy [Figure 3], and coherent anti-Stokes Raman scattering (CARS).[5] The Raman spectra that are obtained can be interpreted using statistical, chemical, and morphological methods.

Raman spectroscopy is also called as a “fingerprint” technique because of its sensitivity to the molecular structure of chemicals. Raman spectroscopy identifies the vibrational information that is specific for the chemical bonds of the organic molecules in the range of ^˜^400-1800 cm^−1^. Use of Raman spectroscopy through fiber-optic probes in the fingerprint region is hindered by the intense background signal, which is generated in the fused-silica fibers. This can be overcome by using optical filters, which necessitates the complex and expensive designs. Koljenović *et al*. used of single, unfiltered, optical fiber for remote Raman spectroscopic tissue characterization in the high wavenumber region (2400-3800 cm^−1^) and showed that essentially the same diagnostic information can be obtained in the high wave number region and in the fingerprint region. They suggested that using the high wavenumber region, and a simple piece of standard optical fiber may eliminate the major obstacle of designing and constructing suitable fiber-optic probes in the fingerprint region.[6] In 2005, Santos *et al*. tested several optical fibers with respect to Raman signal background, to determine their suitability for *in vivo* Raman spectroscopy measurements in the high wavenumber region and concluded that the high flexibility of these probes could facilitate the further development of Raman-guided clinical procedures and interventions.[7]

Raman spectroscopy has evolved rapidly into a technology for analyzing the molecular composition of normal and pathological tissues. Raman spectroscopy was used on biological tissues for the first time by Lord and Yu on the structure of lysozyme.[8] Zonios *et al*. highlighted the potential of Raman technique in providing histological information on precancerous lesions of the mucosal linings in real time without the need for tissue removal.[9] Stone and colleagues assessed the feasibility of Raman spectroscopy for early diagnosis of laryngeal malignancy and showed prediction sensitivities of 83%, 76%, and 92% and specificities of 94%, 91%, and 90% for normal, dysplastic, and squamous cell carcinoma of the larynx, respectively.[10] Promising results of optical signals have been reported in the literature for the diagnosis of Barrett's esophagus, oral cavity lesions, skin cancer, cervical intraepithelial neoplasia, and bladder cancer. [11–14]

Lorincz and colleagues showed that the Raman spectra of the animal samples of kidney, liver, and lung are distinctly different in the intensity distribution of the Raman peaks. They demonstrated that fibrotic tissue has a greater concentration of carotenoids and viable tissue was rich in proteins and nucleic acids. They emphasized the potential use of Raman spectroscopy in clinical diagnosis.[15] Stone *et al*. evaluated Raman spectroscopy for tissue measurements; to target potential malignancies with a clinical need for diagnostic improvements (oesophagus, colon, breast, and prostate) and to build and test spectral libraries and prediction algorithms for tissue types and pathologies. They demonstrated high levels of discrimination between pathologic groups (>90% sensitivity and specificity for all tissues).[16]

## IN UROLOGY

In last few years, there has been an increasing interest in the use of Raman spectroscopy in urology. As Raman spectrum can be obtained in few seconds, it could be combined with endoscopic and laparoscopic procedures without much delay. The literature available to date mainly relates to *in vitro* studies carried out on bladder and prostatic tissue with few articles on *in vivo* studies.

### Bladder

In 1995, Raman spectra were recorded for the first time by Feld and colleagues from the bladder. They showed that bladder cancer has a greater nucleic acid content and lower lipid content than normal bladder urothelium.[17] With this background, Stone *et al*. analysed 196 spectra *in vitro* from bladder samples encompassing normal urothelium, carcinoma *in situ* (CIS), G1, G2, and G3 transitional cell carcinoma (TCC) to construct a diagnostic algorithm. These algorithms when tested with bladder spectra that had not been used in its construction gave excellent sensitivity and specificity.[18] Crow and colleagues are continuing their *in vitro* work to expand the model to include cystitis and in addition trying to develop *in vivo* probes, capable of fitting down the working channel of a cystoscope. These will aid the clinician in making immediate decisions during cystoscopy.

**Figure 4:**
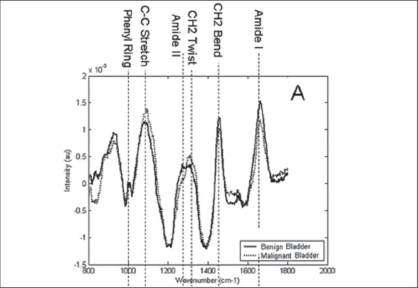
Mean, preprocessed spectra for bladder with major peak assignments [Reprinted from [27], Copyright (2005), with permission from Elsevier]

Raman spectroscopy has been successfully used to differentiate the layers of the urinary bladder wall *in vitro*. Urothelium, lamina propria, and muscle layers could be clearly distinguished based on Raman spectra. Lamina propria spectra were dominated by signal contributions of collagen and the smooth muscle layer showed strong signal contributions of actin. The urothelium had a relatively strong lipid signal contribution. De Jong *et al*. predicted that this technology can be applied *in vivo* by thin, flexible fiberoptic catheters for analysis of the molecular composition of the normal and pathological bladder without the need for biopsies.[19] This kind of information may also aid the urologist to determine the necessary depth of resection for bladder tumor to be carried out. A three-group algorithm constructed by Raman spectroscopy differentiated normal bladder, cystitis, and TCC/CIS with >90% sensitivity and specificity. In addition, this could accurately characterize TCC into low (G1/G2) or high (G3) grade and superficial (pTa) or invasive (pT1/pT2) stage [Figure 4]. It has the potential to provide immediate pathological diagnoses during transurethral resection of bladder tumors.[20]

*In vivo* investigations with optical fiber probes are promising, but generally limited to easily accessible organs. Motz *et al*. implemented an optical design strategy by characterizing the Raman distribution from tissue. This scheme optimizes collection efficiency, minimizes noise, and has resulted in small-diameter, highly efficient Raman probes that are capable of collecting high-quality data within a second. They demonstrated that this new design can advance Raman spectroscopy as a clinically practical technique.[21] In 2005, same group for the first time devised an instrument for *in vivo* tissue analysis, which is capable of collecting and processing Raman spectra in <2 s. This real-time capability of the system was demonstrated *in vivo* during femoral bypass and breast lumpectomy surgeries. There is no doubt that such system will greatly facilitate the adoption of Raman spectroscopy into urological practice, especially for endoscopic diagnosis and interventions, as well as laparoscopic procedures.[22]

Raman molecular imaging (RMI) technology permits visualization of the physical architecture and molecular environment of a cytologic urine sample. Shapiro and colleagues demonstrated that RMI can identify and distinguish between bladder cancer cells and normal bladder cells in voided urine with very high accuracy; and it should be used in screening the high-risk population.[23] Later on, they used a software algorithm and Raman spectroscopy to rapidly identify malignant cells in voided urine and emphasized that RMI of cells in voided urine can accurately distinguish benign from malignant pathology.[24]

### Prostate

**Figure 5:**
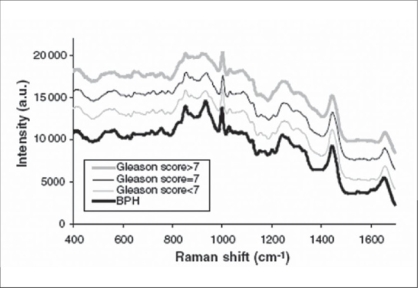
Raman spectra recorded from BPH and prostate cancer grades. [Reprinted from^[26]^, with permission from Macmillan Publishers Ltd.]

Crow *et al*. were the first to describe the use of Raman spectroscopy in prostatic disorders. Raman spectra obtained from the prostate suggested that there was variation in the glycogen and nucleic acid content between benign prostatic hypertrophy (BPH) and adenocarcinoma.[25] A diagnostic algorithm of the prostate confirmed that the technique can accurately differentiate between BPH and prostatic adenocarcinoma *in vitro* [Figure 5].[26] As Raman spectroscopy examines tissue at the molecular level, it has the potential to provide additional prognostic information in early prostate cancer. Further, they suggested that the potential *in vivo* applications of the technique could be for guiding prostate biopsy procedures and the intraoperative assessment of tumor resection margins during radical prostatectomy. *In vitro*, Raman spectroscopy can be used to accurately identify BPH and three different grades of prostatic adenocarcinoma. The prostate algorithm was able to differentiate benign samples (benign prostatic hyperplasia and prostatitis) from prostate cancer, with an overall accuracy of 86%. As Raman probe is suitable for use during endoscopic, laparoscopic, or open procedures, this work paves the way for *in vivo* studies.[27]

Two prostatic cell lines, immortalized normal prostate cell line and malignant cell line derived from prostate metastases were mapped using Raman microscopy by Taleb *et al*. and demonstrated different spectral processing methods data can identify subtle differences between benign and malignant prostatic cells *in vitro*.[28] Raman spectra were measured from two well-differentiated, androgen-sensitive cell lines and two poorly differentiated, androgen-insensitive cell lines to differentiate between carcinoma prostate cell lines of varying degrees of biological aggressiveness. Crow *et al*. were able to identify the individual cell lines with an overall sensitivity of 98% and a specificity of 99% and demonstrated promising results of Raman spectroscopy in the diagnosis and grading of carcinoma of the prostate.[29] Grubisha *et al*. demonstrated the low-level and simultaneous determination of many complexed forms of prostate specific antigen using surface-enhanced Raman scattering (SERS).[30] This technology in future may aid in diagnosis of prostate cancer by analyzing a sample of blood for cancer-specific isoforms of Prostate Specific Antigen (PSA). Using Kerr-gated Raman spectroscopy, Prieto and colleagues were able to obtain spectra from different depths through both the prostate gland and the bladder. They demonstrated the potential use of Kerr-gated Raman spectroscopy to identify a small focus of adenocarcinoma of the prostate gland in an otherwise benign gland. They also showed that it was possible to accurately stage bladder tumors by assessing the base of the tumor following the resection.[31]

### Renal

Allegrini and Sudlow have used the laser Raman microprobe for the characterization and identification of renal lithiasis.[32,33] Laser microprobe was used to differentiate the monohydrate and the dihydrate forms of calcium oxalate inclusions in tissue sections, a potential adjunct in diagnostic pathology. The combined occurrence of both oxalate structures was confirmed in kidney stone specimens thus emphasizing its potential usage in diagnostic pathology.[34] Premasiri *et al*. analyzed the components in human urine and showed that urea concentration in urine is sufficiently high that normal Raman spectroscopy may be used for its analysis. All other components are in low concentrations requiring the use of SERS methods.[35] In 1992, Hong *et al*. used the near-infrared excitation Fourier-transform Raman spectrometry identify the human calculi with various compositions. Using urine as natural biological medium, they obtained good results for both classical Raman laser and Raman laser fiber optics spectroscopy.[36]

Recently, Parekh *et al*. conducted an *ex vivo* study to identify optical characteristics of various renal tissues to examine the feasibility of the Raman spectroscopy to differentiate between malignant and benign renal tissues. They measured the fluorescence and diffuse reflectance spectra from benign and malignant renal tissues obtained from patients undergoing radical nephrectomy. Results showed that all renal tissues, malignant or benign, contain two primary emission peaks; a strong one at approximately 285 nm excitation (Peak A), and a weak one at approximately 340 nm excitation (Peak B). In malignant tissues, the intensity of peak B was lower than benign tissues. They concluded that it is feasible to differentiate between normal and renal cancer tissue using combined fluorescence and diffuse reflectance spectroscopy in an *ex vivo* setting. It may aid in margin detection and tissue discrimination while performing nephron sparing surgery.[37]

### Testis

**Figure 6:**
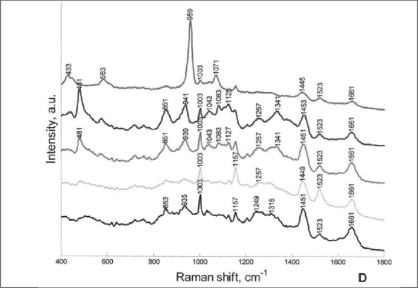
Raman spectra of clusters of testicular parenchyma with microliths [Reprinted from [38], with permission from the American Urological Association]

Testicular microlithiasis is associated with various benign and malignant conditions. Using Raman spectroscopy multiple microliths from samples diagnosed with microlithiasis by ultrasound and subsequently confirmed histologically were investigated. Mapping with Raman spectroscopy demonstrated that these microliths were composed of hydroxyapatite and were located in the seminiferous tubules [Figure 6]. These microliths were found to colocalize with glycogen deposits in germ cell neoplasm.[38]

## FUTURE DIRECTIONS

Further utility and incorporation of Raman spectroscopy, as clinical tool requires additional developments in this technology. First, as most clinical applications of Raman spectroscopy require optical fiber probes, various designs of probes need to be developed to incorporate them into catheters, endoscopes, laparoscopes, cannulas, and needles with ease. Substantial progress is being made in the extraction of morphological information of tissue using Raman spectroscopy. Raman spectroscopy can provide the morphological composition of the tissue in a fraction during a diagnostic procedure or operation and this can bring the insights of pathology into the operating theatre.

Manoharan *et al*. demonstrated the feasibility of Raman spectroscopy to classify accurately normal, benign, and malignant breast tissues and developed needle probes as a tool for improving the accuracy of needle biopsy.[39] Repeat surgeries resulting from positive margins has been reduced following partial mastectomy surgeries using this technology.[40] Such technology will be available for the urologists to determine the margin status intraoperatively when performing oncological operations such as laparoscopic partial nephrectomy and radical prostatectomy. Functional imaging is another exciting field for the use of Raman spectroscopy. Creation of endoscopic images about chemical or morphological properties, and deep tissue Raman imaging may be possible in future. In addition, Raman spectroscopy may be used in combination with ultrasound, computed tomography, and magnetic resonance imaging. Raman tweezers is another exciting field that uses optical tweezers to suspend and manipulate a molecule without direct contact, so that the molecule's Raman spectra may be recorded while it is in its most natural state. The spectra collected with this technique are more reflective of the true nature of the molecule under study as it does not disturb the surrounding environment.[41] This technique may be used in the future to study individual cancer cells *in vitro* and may also provide more insights about host interactions with pathogens in urinary tract infections.

Authors agree with Santos *et al*.[7] that Raman spectroscopy using high wavenumber in fiber optic technology will facilitate the further development of Raman-guided clinical interventions. We completely concur with De Jong *et al*.[19] that *in vivo* applications of Raman spectroscopy through flexible cystoscopy for bladder cancer surveillance can avoid the unnecessary biopsies and can provide immediate pathological diagnosis during resection of bladder tumors. Crow *et al*.[20] have shown the various applications of Raman spectroscopy in prostatic disorders, mainly in differentiation of BPH and adenocarcinoma, its wider use as the sole technology in the future is yet to be defined. We see SERS for analysis of isoforms of PSA from venous blood as the developing technology in diagnosis of prostate cancer. Parekh *et al*.[37] have shown that, *in vitro* use of combined fluorescence and diffuse reflectance spectroscopy to differentiate between normal and renal cancer tissue is possible and the future may see this technology being used in laparoscopic partial nephrectomies.

Raman spectroscopy is a promising clinical tool for real-time diagnosis of disease and evaluation of living tissue *in situ*. The evolution of Raman spectroscopy from earlier visible laser excitation to near-infrared laser excitation and charge-coupled device array detection has amplified its usage in modern medicine. The Raman spectroscopy seems to be a realistic prospect of future due to availability of objective, intraoperative pathological diagnosis.

## CONCLUSION

Raman spectroscopy is an emerging technique that is able to interrogate biological tissues, that gives us an understanding of the changes in molecular structure that are associated with disease development. The potential applications of Raman spectroscopy may herald a new future in the management of various malignant, premalignant, and other benign conditions. Future development and research in Raman spectroscopy may herald a new era for urology.
